# A study on the accessibility and utilisation of targeted drugs for pulmonary arterial hypertension in China

**DOI:** 10.3389/fphar.2026.1671667

**Published:** 2026-01-22

**Authors:** Wei Li, Han Zhang, Lulu Xu, Wan Tang, Wei Lu, Zhengyi You, Huanhuan Wu, Yanquan Lin, Hongdou Chen

**Affiliations:** 1 Department of Pharmacy, The Affiliated Suqian Hospital of Xuzhou Medical University, Suqian, Jiangsu, China; 2 Department of Pharmacy, Nanjing Drum Tower Hospital Group Suqian Hospital, Suqian, Jiangsu, China; 3 School of Pharmacy, Xuzhou Medical University, Xuzhou, Jiangsu, China

**Keywords:** accessibility, affordability, availability, PAH-targeted drugs, utilisation

## Abstract

**Objective:**

This study aimed to evaluate the availability, price levels, affordability, and utilisation of targeted drugs for pulmonary arterial hypertension (PAH) in China.

**Methods:**

This study utilized a retrospective longitudinal design, based on drug procurement data from 859 public hospitals in China, covering the period from January 2019 to December 2023. Accessibility of 11 PAH-targeted therapies was evaluated across three key dimensions: availability (proportion of hospitals stocking the drug), price level (the Defined Daily Dose cost, namely, DDDc = Total annual drug expenditure/DDDs of the drug), and affordability (out-of-pocket expenses as a percentage of household disposable income).

**Results:**

While there was a significant overall improvement in the accessibility of PAH-targeted drugs, structural disparities in accessibility were evident across different drug types, hospital tiers, and between urban and rural areas, as well as among income groups. Availability data showed that tertiary hospitals had significantly better access to NMI-negotiated drugs than secondary hospitals (p = 0.006). Among all drugs, treprostinil had the highest availability (56.1% across hospitals), while newer drugs such as selexipag had very low availability (<10%). Price analysis revealed that the DDDc for most drugs had significantly decreased, including sildenafil (−91.0%) and macitentan (−95.2%), while iloprost remained costly (>3,700 CNY). In terms of affordability, in 2023, all drugs were affordable for the highest-income urban groups, while no drugs were affordable for the lowest-income rural groups, with the cost burden of iloprost accounting for 8,454.8% of the disposable income of the rural population. Drug accessibility exhibited significant structural imbalances, with some drugs being “affordable but hard to obtain” (e.g., riociguat) and others “highly burdensome and poorly accessible” (e.g., treprostinil).

**Conclusion:**

The findings demonstrate significant improvements in the affordability and availability of PAH-targeted therapies over the study period; however, notable inequalities persist in accessibility improvements across urban and rural areas and income groups. Future policies should be tailored to address specific accessibility challenges for different drug categories and focus on overcoming medication access barriers for low-income rural populations to foster health equity.

## 1 Introduction

Pulmonary arterial hypertension (PAH) is a fatal cardiovascular disease characterized by progressive pulmonary vascular remodeling and abnormally elevated pulmonary arterial pressure. It is often referred to as the “cancer of cardiovascular diseases” (Ghofrani et al., 2025). Its onset is associated with multiple factors, including genetics, drug exposure, and environmental toxins (Hassoun, 2021). The global burden of PAH is concentrated in developing countries with limited healthcare resources (accounting for 80%) (Humbert et al., 2023; Global burden of 369 diseases, 2020). China is a high-incidence region for PAH, with 7,300 deaths reported in 2021 (1.82 times the global average) (Wang et al., 2024) and more than 86,000 hospitalized patients in 2022 (GBD, 2021 Pulmonary Arterial Hypertension Collaborators, 2025). Together with India, China bears 33% of the global PAH burden (Yang et al., 2025).

Targeted therapies are the core treatment approach for delaying PAH progression and prolonging survival (Mandras et al., 2020). However, in developing countries, access to pharmacotherapy remains severely challenging due to high drug prices and unequal distribution of healthcare resources (China News Network, 2025). To improve this situation, the Chinese government has implemented a series of measures in recent years: in 2018, idiopathic pulmonary arterial hypertension was included in the First Batch of the Rare Disease List; in 2019, value-added tax reductions were introduced into rare disease drugs; and since 2020, six PAH-targeted drugs have been successively incorporated into the national reimbursement drug list through national medical insurance negotiations (“national negotiation”). Although these measures have effectively improved survival (with the 3-year survival rate increasing from 38.9% to 75.1%), a substantial gap remains between developing and developed countries (Pulmonary Embolism and Pulmonary Vascular, 2021; Luo et al., 2022).

Therefore, systematically evaluating the accessibility of PAH-targeted drugs in China and proposing optimization strategies are of important clinical significance. Such efforts can not only improve patient survival, but also provide evidence to inform improvements in access to rare disease drugs in other resource-limited settings, thereby promoting global health equity.

## 2 Materials and methods

### 2.1 Data sources

This study adopted a retrospective longitudinal design based on drug data from the China Medicine Economic Information (CMEI) database between January 2019 and December 2023. The database covers approximately 1,500 healthcare institutions across 31 provincial-level administrative regions nationwide, which is an authoritative data source for pharmaceutical policy research in China (Science and Technology Development Center of Chinese Pharmaceutical Association, 2025). A total of 859 public general hospitals with complete data quality and continuous reporting were included, comprising 292 secondary hospitals (34%) and 567 tertiary hospitals (66%). Eleven PAH-targeted drugs recommended by the 2022 ESC/ERS Guidelines for the diagnosis and treatment of pulmonary hypertension were selected as study drugs (Table 1). Key variables—generic name, procurement price, and quarterly procurement volume - were systematically extracted from procurement records to establish a comprehensive dataset for evaluating drug accessibility.

**TABLE 1:** Basic information of 11 Pulmonary arterial hypertension (PAH) targeted medications.

Generic name	Dosage form and Strengths (mg)	FDA approval time	China launch time	National medical insurance negotiation	Centralized quantity - based procurement	PAH indication approval time	Generic drug launch time
Sildenafil citrate	Tablet, 25/50/100	1998	2000	—	2020	2020	2014
Tadalafil	Tablet, 5/20	2003	2004	—	2020	Off - label	2019
Vardenafil hydrochloride	Tablet, 10/20	2003	2004	—	—	Off - label	2020
Riociguat	Tablet, 0.5/1/2.5	2013	2017	2020	—	2017	2024
Ambrisentan	Tablet, 5/10	2007	2016	2021	2020	2010	2018
Bosentan	Tablet, 32/125	2001	2006	2020	—	2006	2024
Macitentan	Tablet, 10	2013	2017	2020	—	2017	2023
Beraprost sodium	Tablet, 0.02/0.04	1992^a^	2003	—	—	Off - label	2008
Treprostinil	Injection, 20/50	2005	2013	2023	—	2013	2020
Iloprost	Inhalation solution, 0.02	2004	2006	—	—	2006	—
Selexipag	Tablet, 0.2/0.6/0.8	2015	2018	2020	2024	2018	2024

^a^ stands for “launched in Japan”.

Validity and Limitations of the Data Sourc: Under the current zero-markup drug policy and global budget management for medical insurance, hospital drug procurement behavior is subject to strict institutional constraints, which has created a strong intrinsic linkage between procurement data and actual clinical demand. For specialty drugs used in diseases such as PAH, procurement patterns can effectively reflect healthcare institutions’ service capacity and patients’ real-world medication needs. Thus, procurement data can serve as a valid proxy for assessing drug accessibility. It should be noted that although the nationwide continuous procurement data used in this study cannot fully capture patient-level variations in prescribing behavior, its broad coverage and temporal continuity can provide a robust evidence base for evaluating the impact of pharmaceutical policies on drug accessibility at the macro level. Public general hospitals were selected primarily because of their standardized reporting, continuous time-series data, and central role in PAH diagnosis and long-term management. While this choice may limit the generalizability of the findings, it strengthens internal validity. In addition, the database includes relatively few specialized and primary healthcare institutions, and the procurement volumes of relevant drugs in these settings are minimal.

### 2.2 Evaluation indicator system

Drawing on the standard methodologies of the World Health Organization (WHO) and Health Action International (HAI) (Sisay et al., 2021), this study constructed an evaluation framework across three dimensions.

#### 2.2.1 Availability

Availability was defined as the proportion of the hospitals surveyed that had at least one procurement record for a specific drug during a given quarter. It was calculated as follows:

Availability (%) = (Number of hospitals with drug procurement record/Total number of the hospitals surveyed) × 100%

The availability results were interpreted using an international five-level classification (WHO Collaborating Centr e for Drug Statistics Methodology, 2025):

None: 0% (no supply in any hospital).

Very low: <30% (rare supply in hospitals).

Low: 30%–50% (limited supply in hospitals).

Relatively high: 50%–80% (supply in many hospitals).

High: >80% (supply in most hospitals).

#### 2.2.2 Price level

The price level was measured using the Defined Daily Dose cost (DDDc) as follows:

DDDc (CNY) = Annual out-of-pocket (OOP) expenses per patient/Annual Defined Daily Doses (DDDs).

Where: Annual OOP expenses = Annual drug consumption cost × (1 - Reimbursement Rate).

Annual DDDs = Total annual drug consumption (in mg)/DDD value (in mg).

This study used a median reimbursement rate of 65% to calculate the OOP expenses (Wu). DDD values were preferentially derived from the WHO Anatomical Therapeutic Chemical/Defined Daily Dose (ATC/DDD) system; for drugs without an assigned WHO DDD, their DDD values were determined based on the Chinese Pharmacopoeia (2020 edition) and relevant clinical guidelines (State Food and Drug Administration and National Health Commission, 2025; Chen et al., 2019; Pulmonary Embolism and Pulmonary Vascular Disease Group of the Respiratory Disease Branch of the Chinese Medical Association, Pulmonary Embolism and Pulmonary Vascular Disease Working Committee of the Respiratory Physicians Branch of the Chinese Medical Doctor Association, National Pulmonary Embolism and Pulmonary Vascular Disease Prevention and Treatment Collaboration Group, Expert Group for the Standardized System Construction Project of Pulmonary Hypertension in China, 2021).

#### 2.2.3 Affordability

Affordability was assessed on the basis of the affordability ratio. The formula is: Affordability ratio (%) = (DDDc × 30)/Monthly household disposable income × 100%. Catastrophic health expenditure was used as the evaluation criterion. If the 30-day out-of-pocket treatment cost of a PAH-targeted drug, which can be calculated as (1%–65%) (DDDc×30), exceeded 20% of monthly household disposable income, catastrophic expenditure would be considered to occur, and the drug would be deemed unaffordable (Liu et al., 2021; Wei et al., 2021).

Stratified analysis: To explore differences in economic burden across households with different income levels—especially low-income groups in urban and rural areas—the authors used data on *per capita* disposable income of urban and rural households from the China Statistical Yearbook (National Bureau of Statistics, 2025). Urban and rural households were each divided into five equal groups (income quintiles) from lowest to highest income groups, and affordability ratios were calculated for each quintile. The average household size was assumed to be three persons.

Rationale for reimbursement rate parameter: The baseline reimbursement rate was set at 65%, which was selected based on evidence from multiple sources. Actual reimbursement levels within China’s basic medical insurance system vary by region (e.g., Beijing, Shanghai) and by insurance type (urban employee vs. urban–rural resident schemes), ranging from approximately 40%–90% (National Healthcare Security Administration, 2023; State Council Information Office of China, 2024; Beijing Municipal Medical Security Bureau, 2025; Mu et al., 2022). Published real-world studies on rare disease drugs have further indicated that patients’ actual out-of-pocket shares are mostly concentrated at 10%–30%, corresponding to reimbursement rates of 50%–70% (Che et al., 2024; Chen et al., 2021). Local studies on PAH-targeted drugs have reported similar reimbursement levels (Xin et al., 2016). Therefore, a median value of 65% lies within a reasonable and representative range for this analysis. (Sensitivity analysis: To assess the robustness of affordability estimates to the reimbursement rate parameter, the authors replaced the baseline reimbursement rate (65%) with 40% (lower bound) and 90% (upper bound), repeated the affordability calculation process, and examined affordability ratios for each drug among urban, rural, and overall populations from 2019 to 2023. By comparing results under different scenarios, the authors evaluated the stability of temporal improvement trends and the persistence of urban–rural differences. If the direction of trends and the significance of differences had no change under the two alternative assumptions, conclusions would be considered robust.)

### 2.3 Statistical methods

All indicators related to drug accessibility showed skewed distributions (confirmed by the Shapiro–Wilk normality test). Therefore, all continuous variables were described using medians (interquartile ranges), and between-group comparisons were performed using the Mann–Whitney U test. For comparisons across multiple groups (affordability across income quintiles), the Kruskal–Wallis H test was applied, with Dunn’s post hoc test used for pairwise comparisons where appropriate. All analyses were conducted through SPSS 27.0 and Python 3.9, with a two-sided significance level of α = 0.05.

## 3 Results

### 3.1 Availability

**FIGURE 1:**
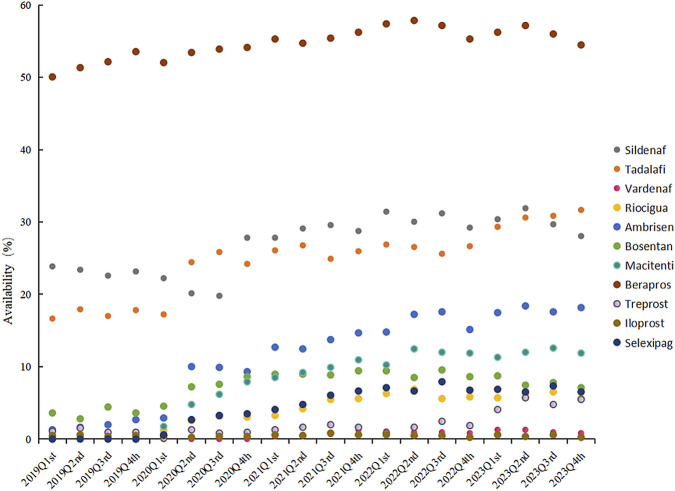
The availability of PAH-targeted drugs (2019–2023).

To evaluate the differences in the availability of PAH - targeted drugs across different hospital tiers and the effect of policy interventions, the authors have analyzed availability data from tertiary and secondary general hospitals from 2019 to 2023. The Mann-Whitney test was used for statistical comparison of the availability differences for 11 PAH-targeted drugs. The results revealed no significant difference in the availability of all 11 drugs between hospital tiers (p > 0.05). However, for the 6 drugs included in the National Reimbursement Drug List (NRDL) negotiations, their availability was significantly higher in tertiary hospitals than in secondary hospitals (p = 0.006). Overall, beraprost sodium maintained the highest availability across all hospitals (51.75%–56.12%), with tertiary hospitals (62.03%–66.43%) consistently showing higher availability than secondary hospitals (30.63%–38.19%). Newer drugs like selexipag (tertiary: 0%–9.43%; secondary: 0%–2.06%) and riociguat (tertiary: 0%–8.25%; secondary: 0%–2.05%) exhibited faster growth trends in tertiary hospitals (details in Table 2; Figure 1). These findings suggest that hospital tiers may be associated with PAH-targeted drug availability, and that national health insurance policy interventions might positively influence the accessibility of specific targeted drugs in tertiary hospitals.

**TABLE 2:** Availability of Pulmonary arterial hypertension (PAH) targeted medications in the investigated hospitals.

No.	Name	All hospitals (%)					Tertiary general hospital (%)					Secondary general hospital (%)				
		2019	2020	2021	2022	2023	2019	2020	2021	2022	2023	2019	2020	2021	2022	2023
1	Sildenafil citrate	23.29	21.19	28.93	30.62	30.04	29.19	26.86	35.47	36.39	36.33	11.32	10.62	15.59	18.98	18.01
2	Tadalafil	17.41	24.33	26.02	26.60	30.74	23.59	31.16	32.47	32.72	37.57	5.14	10.32	12.84	13.31	16.83
3	Vardenafil hydrochloride	0.00	0.00	0.64	0.93	1.11	0.00	0.00	0.65	1.15	1.23	0.00	0.00	0.68	0.68	1.03
4	Riociguat	0.06	2.80	4.83	6.06	6.00	0.00	3.73	5.88	8.02	8.25	0.34	0.00	0.68	2.05	2.05
5	Ambrisentan	1.81	9.16	13.22	16.18	17.87	2.30	10.93	16.25	20.11	22.46	1.03	3.25	5.99	7.02	8.06
6	Bosentan	3.61	7.40	8.96	9.02	7.63	4.94	9.52	12.26	12.35	10.24	1.03	2.57	3.08	3.42	2.57
7	Macitentan	0.00	5.47	9.55	11.64	11.93	0.09	8.76	12.67	15.40	15.08	0.00	1.54	2.89	3.91	5.31
8	Beraprost sodium	51.75	53.67	55.36	57.28	56.12	62.03	63.20	64.65	67.10	66.43	30.63	33.22	35.78	38.19	37.17
9	Treprostinil	0.99	0.87	1.63	1.75	4.77	1.32	1.23	2.18	2.86	6.43	0.34	0.34	0.34	0.17	0.51
10	Iloprost	0.47	0.35	0.58	0.47	0.47	0.71	0.53	0.88	0.71	0.71	0.00	0.00	0.00	0.34	0.34
11	Selexipag	0.00	2.97	5.41	6.93	6.52	0.00	4.41	7.12	9.23	9.43	0.00	0.17	1.71	2.40	2.06

Availability (%) = (Number of hospitals with drug procurement record/Total number of the hospitals surveyed) × 100%.

### 3.2 DDDc

To investigate the potential impact of health insurance policies on the cost of PAH-targeted drugs, this study has analyzed the changes in the Defined Daily Dose cost (DDDc) for 11 drugs from 2019 to 2023. Specifically, sildenafil citrate (51.24 CNY→4.62 CNY, −91.0%) and tadalafil (160.67 CNY→48.48 CNY, −69.8%) showed substantial price reductions after the 2020 volume-based procurement (VBP). Ambrisentan (130.24 CNY→10.97 CNY, −91.6%) demonstrated a significant cost decrease following its inclusion in the 2021 NRDL negotiations. Drugs added to the reimbursement list in 2020, such as macitentan (999.33 CNY→48.29 CNY, −95.2%) and riociguat (280.91 CNY→34.92 CNY, −87.6%), also experienced sharp declines. Treprostinil (2,125.16 CNY→370.66 CNY, −82.6%), newly included in the 2023 NRDL negotiations, began to show a substantial cost-reduction trend. The cost of drugs like beraprost sodium remained relatively stable (36.06 CNY→33.02 CNY), while iloprost (3,641.55 CNY→3,708.84 CNY) maintained a high price level (details in Table 3). The significant reductions in drug costs closely coincided with the implementation of relevant health insurance policies, suggesting that VBP and NRDL negotiations may have played a positive role in reducing the daily treatment cost of PAH-targeted drugs. However, the cost optimization of some high-priced drugs still needs to call for further attention.

**TABLE 3:** DDDc of Pulmonary arterial hypertension (PAH) targeted medications in the investigated hospitals.

No.	Name	DDD (mg)	DDDc (CNY)				
			2019	2020	2021	2022	2023
1	Sildenafil citrate	60.00	51.24	19.21	7.06	5.42	4.62
2	Tadalafil	30.00	160.67	95.46	74.23	75.41	48.48
3	Vardenafil hydrochloride	10.00	NA	NA	34.63	34.83	35.13
4	Riociguat	4.50	280.91	34.04	34.36	34.30	34.92
5	Ambrisentan	7.50	130.24	39.71	17.45	11.78	10.97
6	Bosentan	250.00	139.29	37.86	38.32	38.44	38.35
7	Macitentan	10.00	999.33	48.30	48.30	48.30	48.29
8	Beraprost sodium	0.20	36.06	35.74	35.20	34.75	33.02
9	Treprostinil	4.30	2,125.16	2,141.76	2,139.16	2,116.01	370.66
10	Iloprost	0.15	3,641.55	3,672.49	3,706.27	3,824.87	3,708.84
11	Selexipag	1.80	NA	153.88	152.14	133.09	134.16

DDDc , Total annual drug expenditure/DDDs, of the drug.

### 3.3 Affordability

From 2019 to 2023, the medicine expenditure for all drugs generally showed a downward trend in both urban and rural areas, but a significant urban-rural gap persisted (p < 0.05) (detials in Table 4). Although the gap narrowed as income increased, low-income groups, especially in rural areas, consistently suffered from a higher financial burden from drug costs. Specific findings are as follows:

**TABLE 4:** Monthly drug cost as percentage of household capacity-to-pay in China. (under 65% reimbursement scenario).

​	Total (%)					Urban (%)					Rural (%)				
Name	2019	2020	2021	2022	2023	2019	2020	2021	2022	2023	2019	2020	2021	2022	2023
Sildenafil citrate	**20.04**	7.2	2.4	1.8	1.44	14.52	5.28	1.8	1.32	1.08	**38.4**	13.44	4.44	3.24	2.52
Tadalafil	**62.76**	**35.64**	**25.32**	**24.48**	14.88	**45.48**	**26.16**	18.84	18.36	11.28	**120.36**	**66.84**	**47.04**	**45**	**26.88**
Vardenafil hydrochloride	NA	NA	11.88	11.28	10.8	NA	NA	8.76	8.52	8.16	NA	NA	21.96	20.76	19.44
Riociguat	**109.68**	12.72	11.76	11.16	10.68	**79.56**	9.36	8.64	8.4	8.04	**210.36**	**23.88**	**21.72**	**20.4**	19.32
Ambrisentan	**50.88**	14.76	6	3.84	3.36	**36.84**	10.92	4.44	2.88	2.52	**97.56**	**27.84**	11.04	7.08	6.12
Bosentan	**54.36**	14.16	13.08	12.48	11.76	**39.48**	10.32	9.72	9.36	8.88	**104.28**	**26.52**	**24.24**	**22.92**	**21.24**
Macitentan	**390.24**	18	16.44	15.72	14.76	**283.08**	13.2	12.24	11.76	11.16	**748.56**	**33.84**	**30.6**	**28.8**	**26.76**
Beraprost sodium	14.04	13.32	12	11.28	10.08	10.2	9.84	8.88	8.52	7.68	**27**	**25.08**	**22.32**	**20.76**	18.24
Treprostinil	**829.8**	**798.48**	**730.8**	**688.44**	**113.4**	**602.04**	**586.32**	**541.44**	**515.28**	85.8	**1,591.8**	**1,500.24**	**1,356**	**1,261.2**	**205.08**
Iloprost	**1,421.88**	**1,369.08**	**1,266.12**	**1,244.4**	**1,134.84**	**1,031.64**	**1,005.36**	**938.04**	**931.32**	**858.84**	**2,727.6**	**2,572.56**	**2,349.36**	**2,279.76**	**2051.88**
Selexipag	NA	**57.36**	**51.96**	**43.32**	**41.04**	NA	**42.12**	**38.52**	**32.4**	**31.08**	NA	**107.76**	**96.48**	**79.32**	**74.16**

NA, not available. Bold values indicate that the monthly drug cost exceeds 20% of household capacity-to-pay, representing catastrophic health expenditure.

Highest 20% income group: In urban areas, affordability improved from only two drugs affordable (affordability ratio <20%) in 2019 to all drugs affordable in 2023. In rural areas, affordability improved from no affordable drugs in 2019 to a situation in 2023 where only iloprost (887.71%) and treprostinil (88.72%) exceeded the catastrophic expenditure threshold (20%), with all other drugs meeting the affordability standard. The urban-to-rural burden ratio was 2.0–2.2:1. (Supplementary Table S4A).

Middle-high 20% income group: In 2023, only three high-cost drugs (iloprost, treprostinil, and selexipag) exceeded the threshold in urban areas. In rural areas, seven drugs met the affordability standard, while iloprost (1,713.02%) and treprostinil (171.20%) remained at extremely high burden levels. The urban-to-rural burden ratio was 2.0–2.3:1. (Supplementary Table S4B).

Middle 20% income group: Affordability in urban areas improved from no affordable drugs in 2019 to a situation in 2023 where only iloprost, treprostinil, and selexipag exceeded the threshold. In rural areas, five drugs were affordable. The burden for iloprost (2,408.47%) and treprostinil (240.70%) in rural areas was 2.5 times higher than that in urban areas, indicating a widening gap. (Supplementary Table S4C).

Middle-low 20% income group: In 2023, five drugs were affordable in urban areas, with selexipag (49.99%) nearing the threshold (<50%). In rural areas, only three drugs were affordable. The overall urban-to-rural burden ratio across all drug categories was 2.1–2.4:1. The burden ratio for iloprost was as high as 3,459.74%, far exceeding the threshold. (Supplementary Table S4D).

Lowest 20% income group: In 2023, four drugs met the affordability standard in urban areas, while selexipag (92.11%) and the two high-cost drugs (iloprost and treprostinil) exceeded the threshold. In rural areas, no drugs were affordable throughout the period from 2019 to 2023. In 2023, the burden for iloprost (8,454.81%) and treprostinil (844.98%) in rural areas was 3.3 times that in urban areas, representing the most severe economic burden. (Supplementary Table S4E).

**FIGURE 2:**
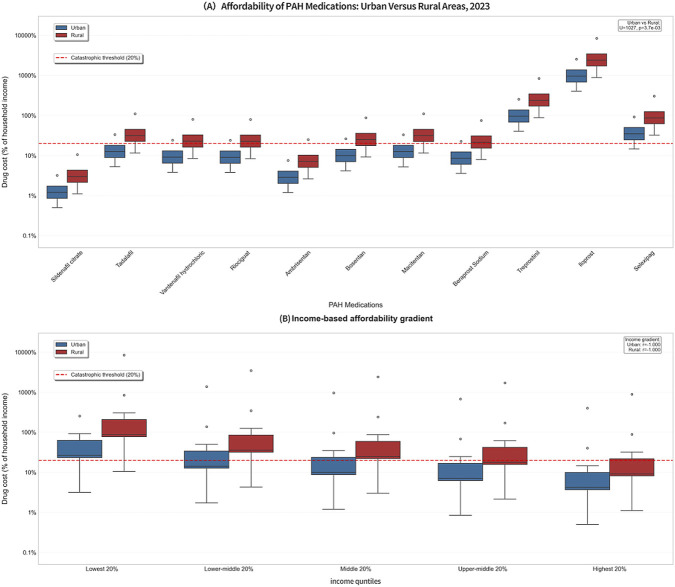
Affordability of PAH medications by residence and income level, 2023. **(A)** Comparison of drug cost as a percentage of household income between urban (blue) and rural (red) areas for various PAH medications. **(B)** Income-based affordability gradient, showing the drug cost burden across population income quintiles for urban and rural residents. In both panels, the dashed horizontal line indicates the catastrophic expenditure threshold (20% of household income). Data are presented as box plots.

This study created box plots of the affordability ratios (Figure 2) to visually present the overall distribution and dispersion of drug affordability across different income strata and between urban and rural areas in 2023. These plots comprehensively display the central tendency, variability, and outliers of medicine expenditure burden for urban and rural residents within the five income groups. They clearly reveal a “dual imbalance” pattern: overall affordability worsens as income decreases, and the burden is systematically higher in rural areas than in urban areas across all income levels.

### 3.4 Comprehensive analysis of affordability and availability

**FIGURE 3:**
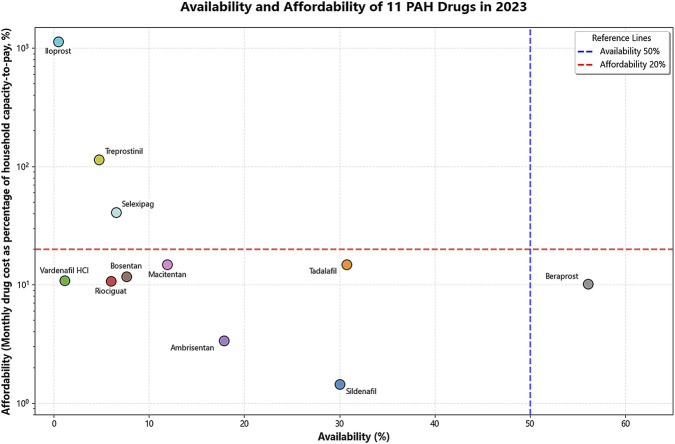
Availability and affordability of 11 PAH drugs in 2023.

In 2023, a significant structural imbalance was observed between the affordability and availability of the 11 drugs. Treprostinil and iloprost were characterized by “high burden and low accessibility”, with extremely high affordability indices (1,134.84 and 113.4, respectively) and very low availability rates (0.47% and 4.77%, respectively). Another group of drugs, including sildenafil citrate, tadalafil, riociguat, ambrisentan, bosentan, and macitentan, showed a pattern of “affordable but difficult to obtain”. These drugs demonstrated relatively favourable affordability (ranging from 1.44 to 14.88), but their availability rates in hospitals were all below 31%, among which riociguat and bosentan had availability rates below 10%. Beraprost sodium was the only drug showing relative balance in both metrics, with an availability rate of 56.12% and an affordability index of 10.08 (Figure 3).

In this study, for drugs included in the NRDL, an average reimbursement rate of 65% was assumed to estimate patients’ out-of-pocket expenses. To test the robustness of this assumption, sensitivity analyses were performed by using reimbursement rates of 40% and 90%. It is important to note that among the 11 drugs studied, as of 2023, only six—riociguat, ambrisentan, bosentan, macitentan, treprostinil, and selexipag—were included in the NRDL, indicating that their out-of-pocket costs are affected by the reimbursement rate. The sensitivity analyses confirmed that, although the absolute level of out-of-pocket costs varied with the reimbursement rate, the core conclusions of this study remained robust under all scenarios. These core conclusions can be summarized as the overall improvement trend in drug affordability, the persistent imbalance in accessibility across different drug categories, and the significant and sustained urban-rural disparity. (Supplementary Tables S5A,S5B).

## 4 Discussion

### 4.1 Study limitations

To the best of its knowledge, this study represents the first systematic assessment of the accessibility of targeted therapies for PAH in China. Compared with 2019, both the overall availability and affordability of the 11 PAH-targeted drugs had improved substantially by 2023. Macitentan, bosentan, selexipag, and riociguat were included in the NRDL through national price negotiations in 2020, while sildenafil, tadalafil, and ambrisentan were incorporated into the national centralized procurement program in the same year. The findings indicate that within 1 year upon policy implementation, the availability of these drugs increased markedly.

Among all the evaluated drugs, beraprost sodium demonstrated the highest availability, with a relatively stable but sustained upward trend. This pattern can be attributed to its early market entry (approved in 2006), broad indications (covering both PAH and chronic arterial occlusive diseases), and dominant market share, with domestic manufacturers accounting for approximately 80%–85% of the market, collectively contributing to price stability (Popular generic drug reviews, 2025; Peng et al., 2024). Previous studies have suggested that earlier market entry facilitates clinical adoption and hospital formulary inclusion (Chen and Feng, 2024). Availability was significantly higher in tertiary hospitals, possibly reflecting patient care-seeking preferences and institutional drug-stocking practices (Zhu et al., 2023). Evidence indicates that healthcare accessibility and technological capacity are the primary determinants of the influence in patients’ hospital choice (Wu, 2025). Data from the Hospital Quality Monitoring System showed that in 2022, tertiary hospitals admitted 824,000 PAH inpatients, compared with 306,000 in secondary hospitals (Overview of the medical quality, 2024). Reports from the China Pharmaceutical Industry Association in 2022 further confirmed that tertiary hospitals are the main settings for the utilization of negotiated drugs (Wu et al., 2025; China Association of Enterprises with Foreign Investment Drug Research and Development Industry Committee, 2025).

The results of the study also revealed a lag in the clinical use of vardenafil in China. Although the originator product was launched in China in June 2004, its first generic version did not enter the market until July 2020. In this study, vardenafil was unavailable in the hospitals surveyed between 2019 and 2020, and its generic products only began to appear in 2021. This delay may be attributable to the absence of regulatory approval for its PAH indication in China, which has constrained standardized clinical use of vardenafil. In addition, compared with the relatively high price of the originator drug (82 CNY for 5 mg, 98 CNY for 10 mg, and 128 CNY for 20 mg), vardenafil lacks price competitiveness relative to sildenafil (Ruopp and Cockrill, 2022). Pharmacoeconomic studies have demonstrated that sildenafil and tadalafil have lower incremental cost-effectiveness ratios than apomorphine (Zhao et al., 2022). Furthermore, the inclusion of sildenafil and tadalafil in the national centralized procurement program in 2020, followed by substantial price reductions, was likely to further marginalize vardenafil in clinical practice.

With respect to pricing, compared with 2019, the DDDc of all drugs decreased significantly by 2023, with reductions exceeding 90% for macitentan, ambrisentan, and sildenafil. The price of treprostinil remained stable prior to 2023; however, following its inclusion in the NRDL through national negotiations in March 2023, its DDDc declined by 82.48%, consistent with findings from previous studies (Zou et al., 2023; Ren et al., 2023). Two earlier studies also reported that centralized procurement policies substantially reduced the prices of most cardiovascular drugs, thereby improving patient affordability (Chen, 2023; Zhang et al., 2022). In contrast, iloprost, a prostacyclin analog still under patent protection and without policy support, maintained a high DDDc of approximately 3,700 CNY.

Based on analyses across five income groups, the authors found that rural residents systematically suffered from a higher drug cost burden than urban residents within the same income strata, with this disparity expanding exponentially as income decreased. For example, in the highest income group in 2023, the urban–rural burden difference for iloprost was 402.26% versus 887.71% (a gap of 485.45%); in the lowest income group, this disparity surged to 2,546.41% versus 8,454.81% (a gap of 5,908.4%). These findings suggest that low income and rural household registration exert a synergistic amplifying effect rather than a simple additive one.

From the demand-side perspective, fragmentation of health insurance reimbursement policies constitutes a core driver of patients’ financial burden. Although multiple PAH-targeted therapies have been included in the NRDL, substantial regional heterogeneity exists in the real-world implementation of outpatient special disease (OSD) coverage. For instance, Yunnan Province has established a relatively comprehensive outpatient reimbursement mechanism for negotiated drugs, under which PAH patients covered by resident insurance can receive reimbursement at a rate of 70% (Yunnan Provincial Healthcare Security Administration, 2025). However, in other regions, gaps between policy design and implementation persist. In practice, core PAH therapies and adjunctive medications used to manage complications or adverse effects may be classified under different reimbursement channels with separate deductibles, thereby increasing patients’ out-of-pocket expenses. Such uneven implementation of OSD policies directly undermines the economic feasibility of sustained and standardized treatment.

Structural disparities within the medical insurance system further exacerbate inequity. Overall, reimbursement rates for inpatient services under the Urban–Rural Resident Basic Medical Insurance are generally lower than those under the Urban Employee Basic Medical Insurance. When combined with the typically lower income levels of rural residents, this discrepancy generates a pronounced “income–hukou (i.e., household register)” dual disadvantage, rendering rural patients particularly vulnerable to catastrophic health expenditures for chronic conditions requiring long-term medication, such as PAH. However, this “geo-economic” dual inequality is not unique to China. The World Health Organization reports that more than half of the global population lacks access to essential health services due to geographic or financial barriers, and that residents of low-income countries face more than threefold higher risks of catastrophic medical spending than those in high-income countries (World Health Organization, 2021).

This study further demonstrated that as income levels decline, the proportion of drug costs relative to payment capacity increases in an almost exponential manner. For example, in 2023, the burden of tadalafil among urban patients rose more than sixfold from 5.26% in the highest income group to 33.29% in the lowest income group. This non-linear trend underscores the dominant role of income in shaping drug accessibility. As income decreases, particularly among rural patients, affordable therapeutic options diminish sharply. While patients in the highest income group may still retain multiple treatment choices, those in the lowest income group approach virtually no targeted therapy affordable, resulting in a severe “accessibility gap”. These findings are consistent with those of Frost et al., who reported that in resource-constrained settings, the marginal impact of income on medicine accessibility increases dramatically as economic conditions worsen (Frost and Reich, 2009).

This study identified a classic pattern of “structural imbalance” in the accessibility of PAH-targeted therapies. Specifically, some drugs, such as certain prostacyclin analogs, are included in hospital formularies but remain unaffordable for patients, causing that “drugs are available in hospitals but unaffordable for patients”. Conversely, other drugs, such as bosentan, have become financially accessible following national price negotiations, but yet remain unavailable due to insufficient hospital stocking, creating a scenario where “patients can afford the drugs, but hospitals fail to provide them”. This imbalance reflects inadequate coordination between the supply and demand sides of the healthcare system.

From the demand side, the “available but unaffordable” problem primarily stems from deficiencies in the medical insurance system, including fragmented benefit packages, inadequate risk-sharing mechanisms for high-cost rare disease drugs, and institutional constraints on the use of expensive drugs under payment reform, which indirectly increase the cost-sharing proportion of patients. From the supply side, the “affordable but unavailable” issue highlights implementation bottlenecks, such as misalignment between the NRDL and essential drugs lists, inconsistencies in formularies across hospital tiers, and dual challenges in drug supply and insurance settlement under the “dual-channel” policy at the primary care level.

To address this structural dilemma, a precisely coordinated governance framework is required. On the demand side, multi-tiered protection mechanisms should be strengthened through provincially pooled special funds and the development of inclusive commercial insurance. On the supply side, hospital incentive structures should be reformed, performance evaluation metrics optimized, and “green channels” for essential drug procurement established. At the system level, deeper integration of the “three-medicine linkage” reform (medical services, medical insurance, and pharmaceuticals) is needed, alongside value-based payment standards, strengthened regional medical centers, and improved drug availability monitoring mechanisms. Only through coordinated reforms on both the supply and demand sides can institutional barriers and policy fragmentation be overcome, enabling a substantive transition from “drug availability” to “effective access” and ensuring equitable access to quality treatment.

The imbalance in access to PAH therapies fundamentally reflects deeper structural tensions within the healthcare system. From an international comparative perspective, insufficient access to PAH-targeted drugs is a common challenge worldwide, but some countries operating under different healthcare systems have developed diverse policy responses that can offer valuable lessons for China. Countries with universal healthcare systems, such as Australia, control the costs of high-value drugs through health technology assessment (HTA) and risk-sharing mechanisms (Tamura et al., 2017). In the United Kingdom, the National Institute for Health and Care Excellence (NICE) applies cost-effectiveness thresholds (typically £20,000–£30,000 per QALY) to guide value-based reimbursement decisions for PAH-targeted drugs (Services Australia, 2021). Social health insurance systems in Germany and Japan link reimbursement to disease management programs (DMPs), whereby drugs such as bosentan must be prescribed and monitored within designated specialist centers (Guan et al., 2015; Zhu and Yang, 2021).

Experience from low- and middle-income countries under resource constraints is particularly instructive. Thailand has substantially improved PAH treatment accessibility through dynamic adjustment of its national drug list and pro-generic policies (Teerawattananon et al., 2014), while India has leveraged domestic pharmaceutical capacity to markedly reduce the prices of drugs such as iloprost (IMS Health, 2025). These cases suggest that tiered coverage strategies may represent a more sustainable approach under limited-resource conditions. In addition, various countries and regions have established targeted financing mechanisms for rare disease drugs: Canada and South Korea have created dedicated rare disease funds (Health Canada, 2025; Ng et al., 2024); and Taiwan has achieved long-term payment stability for orphan drugs through diversified financing and earmarked accounts (Hsu et al., 2018). The United States National Health Service has promoted value-based agreements (VBAs), linking payment to real-world outcomes, such as performance-based reimbursement for macitentan, to reduce ineffective expenditures (Cole et al., 2021).

Drawing on these international experience and China’s contextual realities, policy optimization can be pursued along three dimensions. First, a tiered protection framework should be established to ensure full coverage for essential drugs, set out-of-pocket caps for moderately priced drugs, and explore provincial or national special funds for ultra-high-cost therapies. Second, payment reforms should be advanced through outcome-based risk-sharing agreements and cross-regional joint procurement to reduce price levels and payment uncertainty. Third, supportive institutional arrangements should be strengthened by incorporating PAH-targeted drug accessibility into hospital performance assessments and enhancing tiered diagnosis and treatment systems alongside “dual-channel” supply management. Furthermore, a national inter-ministerial coordination mechanism for rare disease drug coverage should be established to strengthen collaboration among health insurance, regulatory, and health authorities, which can be supported by dynamic monitoring and evaluation based on real-world data to ensure continuous policy refinement.

Despite its rigor, this study has several inherent limitations. First, at the data level, hospital procurement data were used as a proxy for drug accessibility. Although procurement data are closely linked to clinical demand under the zero-markup policy and effectively capture macro-level trends, they cannot precisely reflect patient-level prescribing and medication use. While sensitivity analyses confirmed the robustness of our core findings, future studies incorporating anonymized prescription-level or insurance claims data would provide more granular validation. Additionally, due to systematic barriers in accessing pharmacy and specialty hospital data, this study is limited to public general hospitals and may not fully capture medication use in specialized institutions or designated pharmacies, potentially limiting generalizability.

Second, with respect to study scope, the authors did not evaluate combination therapy regimens due to the absence of standardized combination protocols for PAH treatment in China and the evolving heterogeneity of clinical practice. Given that combination therapy is strongly recommended in current guidelines, this represents a key limitation shared by many real-world drug accessibility studies. These limitations largely reflect systemic constraints in research conditions and data infrastructure rather than shortcomings specific to this study.

## 5 Conclusion

The findings demonstrate significant improvements in the affordability and availability of PAH-targeted therapies over the study period, closely aligned with the implementation timelines of national price negotiation and centralized procurement policies. However, these improvements are characterized by pronounced structural imbalances, manifested as the compounding effects of urban–rural disparities and income gradients. Rural residents and low-income populations continue to face substantial barriers to treatment access. Future policy efforts should prioritize resolving the coexistence of “affordable but unavailable” and “available but unaffordable” drugs by strengthening primary-level drug supply, improving insurance reimbursement coordination, and advancing targeted protection mechanisms, so as to enhance the equity and sustainability of access to PAH therapies.

## Data Availability

Publicly available datasets were analyzed in this study. This data can be found here: https://www.pharnexcloud.com/.
