# Supplementary material for: A study on the accessibility and utilisation of targeted drugs for pulmonary arterial hypertension in China
Source: Front Pharmacol. 2026 Jan 22;17:1671667. doi: 10.3389/fphar.2026.1671667 (PMC12873474; doi:10.3389/fphar.2026.1671667)
Supplement: Supplementary file 1 [file Supplementaryfile1.doc]

**Supplementary Table 1** Quarterly availability of Pulmonary arterial hypertension (PAH) targeted drugs in all investigated hospitals from 2019 to 2023.

| **No.** | **Name** | **2019 (%)** | | | |  | **2020 (%)** | | | |  | **2021 (%)** | | | |  | **2022 (%)** | | | |  | **2023 (%)** | | | |
| --- | --- | --- | --- | --- | --- | --- | --- | --- | --- | --- | --- | --- | --- | --- | --- | --- | --- | --- | --- | --- | --- | --- | --- | --- | --- |
|  |  | Q1st | Q2nd | Q3rd | Q4th |  | Q1st | Q2nd | Q3rd | Q4th |  | Q1st | Q2nd | Q3rd | Q4th |  | Q1st | Q2nd | Q3rd | Q4th |  | Q1st | Q2nd | Q3rd | Q4th |
| 1 | Sildenafil citrate | 23.86 | 23.4 | 22.58 | 23.17 |  | 22.24 | 20.14 | 19.79 | 27.82 |  | 27.82 | 29.1 | 29.57 | 28.75 |  | 31.43 | 30.03 | 31.2 | 29.22 |  | 30.38 | 31.9 | 29.69 | 28.06 |
| 2 | Tadalafil | 16.65 | 17.93 | 17 | 17.81 |  | 17.23 | 24.45 | 25.84 | 24.21 |  | 26.08 | 26.78 | 24.91 | 25.96 |  | 26.89 | 26.54 | 25.61 | 26.66 |  | 29.34 | 30.62 | 30.85 | 31.66 |
| 3 | Vardenafil hydrochloride | 0.00 | 0.00 | 0.00 | 0.00 |  | 0.00 | 0.00 | 0.00 | 0.00 |  | 0.35 | 0.58 | 0.70 | 1.28 |  | 1.05 | 0.93 | 0.93 | 0.81 |  | 1.28 | 1.28 | 0.93 | 0.81 |
| 4 | Riociguat | 0.12 | 0.12 | 0.00 | 0.00 |  | 1.16 | 2.56 | 3.14 | 3.03 |  | 3.26 | 4.19 | 5.47 | 5.59 |  | 6.29 | 6.87 | 5.59 | 5.82 |  | 5.70 | 6.29 | 6.52 | 5.59 |
| 5 | Ambrisentan | 1.28 | 1.63 | 1.98 | 2.68 |  | 2.91 | 10.01 | 9.90 | 9.31 |  | 12.69 | 12.46 | 13.74 | 14.67 |  | 14.78 | 17.23 | 17.58 | 15.13 |  | 17.46 | 18.39 | 17.58 | 18.16 |
| 6 | Bosentan | 3.61 | 2.79 | 4.42 | 3.61 |  | 4.54 | 7.22 | 7.57 | 8.61 |  | 8.96 | 8.96 | 8.85 | 9.43 |  | 9.43 | 8.50 | 9.55 | 8.61 |  | 8.73 | 7.45 | 7.80 | 7.10 |
| 7 | Macitentan | 0.00 | 0.12 | 0.00 | 0.00 |  | 1.75 | 4.77 | 6.17 | 7.92 |  | 8.50 | 9.20 | 9.90 | 10.94 |  | 10.24 | 12.46 | 11.99 | 11.29 |  | 11.29 | 11.99 | 12.57 | 11.87 |
| 8 | Beraprost Sodium | 50.06 | 51.34 | 52.15 | 53.55 |  | 52.04 | 53.43 | 53.90 | 54.13 |  | 55.30 | 54.71 | 55.41 | 56.23 |  | 57.39 | 57.86 | 57.16 | 55.30 |  | 56.23 | 57.16 | 56.00 | 54.48 |
| 9 | Treprostinil | 1.05 | 1.51 | 0.93 | 0.93 |  | 0.12 | 1.28 | 0.81 | 0.93 |  | 1.28 | 1.63 | 1.98 | 1.63 |  | 0.81 | 1.63 | 2.44 | 1.86 |  | 4.07 | 5.70 | 4.77 | 5.47 |
| 10 | Iloprost | 0.47 | 0.58 | 0.35 | 0.47 |  | 0.47 | 0.23 | 0.35 | 0.35 |  | 0.58 | 0.47 | 0.81 | 0.58 |  | 0.58 | 0.47 | 0.47 | 0.23 |  | 0.58 | 0.35 | 0.58 | 0.23 |
| 11 | Selexipag | 0.00 | 0.00 | 0.00 | 0.00 |  | 0.58 | 2.68 | 3.26 | 3.49 |  | 4.07 | 4.77 | 6.05 | 6.64 |  | 7.10 | 6.64 | 7.92 | 6.75 |  | 6.87 | 6.52 | 7.33 | 6.52 |

**Supplementary Table 2** Quarterly availability of Pulmonary arterial hypertension (PAH) targeted drugs in tertiary general hospitals from 2019 to 2023.

| **No.** | **Name** | **2019 (%)** | | | |  | **2020 (%)** | | | |  | **2021 (%)** | | | |  | **2022 (%)** | | | |  | **2023 (%)** | | | |
| --- | --- | --- | --- | --- | --- | --- | --- | --- | --- | --- | --- | --- | --- | --- | --- | --- | --- | --- | --- | --- | --- | --- | --- | --- | --- |
|  |  | Q1st | Q2nd | Q3rd | Q4th |  | Q1st | Q2nd | Q3rd | Q4th |  | Q1st | Q2nd | Q3rd | Q4th |  | Q1st | Q2nd | Q3rd | Q4th |  | Q1st | Q2nd | Q3rd | Q4th |
| 1 | Sildenafil citrate | 29.63 | 29.28 | 28.92 | 29.10 |  | 28.04 | 25.57 | 24.69 | 35.45 |  | 34.74 | 35.8 | 36.16 | 35.10 |  | 37.57 | 35.8 | 37.21 | 35.63 |  | 37.04 | 38.1 | 35.63 | 33.51 |
| 2 | Tadalafil | 22.57 | 23.81 | 23.28 | 24.34 |  | 22.75 | 31.22 | 32.1 | 31.57 |  | 32.45 | 34.39 | 31.22 | 32.63 |  | 34.04 | 32.98 | 31.92 | 33.51 |  | 35.63 | 37.92 | 38.27 | 37.74 |
| 3 | Vardenafil hydrochloride | 0.00 | 0.00 | 0.00 | 0.00 |  | 0.00 | 0.00 | 0.00 | 0.00 |  | 0.53 | 0.53 | 0.88 | 1.59 |  | 1.23 | 1.06 | 1.23 | 0.53 |  | 1.41 | 1.41 | 0.88 | 1.06 |
| 4 | Riociguat | 0.00 | 0.00 | 0.00 | 0.00 |  | 1.76 | 3.70 | 4.76 | 4.59 |  | 4.59 | 6.17 | 7.76 | 7.76 |  | 8.47 | 8.99 | 7.58 | 7.76 |  | 7.76 | 8.47 | 8.82 | 7.05 |
| 5 | Ambrisentan | 1.41 | 1.94 | 2.65 | 3.53 |  | 4.06 | 12.35 | 13.23 | 12.52 |  | 17.11 | 15.7 | 17.81 | 18.69 |  | 18.87 | 21.34 | 22.75 | 19.58 |  | 22.22 | 23.28 | 22.75 | 22.4 |
| 6 | Bosentan | 4.94 | 3.70 | 5.64 | 4.94 |  | 6.17 | 9.52 | 10.23 | 11.46 |  | 12.35 | 12.17 | 11.11 | 12.7 |  | 12.87 | 11.11 | 12.35 | 11.46 |  | 12.17 | 10.05 | 10.41 | 9.88 |
| 7 | Macitentan | 0.00 | 0.18 | 0.00 | 0.00 |  | 2.65 | 7.05 | 8.47 | 11.29 |  | 11.82 | 12.17 | 13.58 | 14.99 |  | 13.93 | 16.93 | 15.87 | 14.99 |  | 14.46 | 15.34 | 16.75 | 15.52 |
| 8 | Beraprost Sodium | 61.02 | 61.73 | 63.32 | 64.2 |  | 62.96 | 63.84 | 64.55 | 63.84 |  | 65.43 | 64.90 | 65.26 | 65.96 |  | 67.20 | 67.37 | 67.02 | 64.9 |  | 66.49 | 67.37 | 65.78 | 62.96 |
| 9 | Treprostinil | 1.41 | 2.12 | 1.23 | 1.23 |  | 0.18 | 1.59 | 1.23 | 1.23 |  | 1.59 | 2.47 | 3.00 | 2.29 |  | 1.06 | 2.47 | 3.70 | 2.82 |  | 5.82 | 8.29 | 7.05 | 7.41 |
| 10 | Iloprost | 0.71 | 0.88 | 0.53 | 0.71 |  | 0.71 | 0.35 | 0.53 | 0.53 |  | 0.88 | 0.71 | 1.23 | 0.88 |  | 0.71 | 0.53 | 0.71 | 0.35 |  | 0.71 | 0.53 | 0.71 | 0.35 |
| 11 | Selexipag | 0.00 | 0.00 | 0.00 | 0.00 |  | 0.88 | 4.06 | 4.76 | 5.11 |  | 5.82 | 6.53 | 7.76 | 8.99 |  | 9.52 | 8.64 | 10.76 | 8.99 |  | 9.17 | 9.17 | 9.7 | 8.99 |

**Supplementary Table 3** Quarterly availability of Pulmonary arterial hypertension (PAH) targeted drugs in secondary general hospitals from 2019 to 2023.

| **No.** | **Name** | **2019 (%)** | | | |  | **2020 (%)** | | | |  | **2021 (%)** | | | |  | **2022 (%)** | | | |  | **2023 (%)** | | | |
| --- | --- | --- | --- | --- | --- | --- | --- | --- | --- | --- | --- | --- | --- | --- | --- | --- | --- | --- | --- | --- | --- | --- | --- | --- | --- |
|  |  | Q1st | Q2nd | Q3rd | Q4th |  | Q1st | Q2nd | Q3rd | Q4th |  | Q1st | Q2nd | Q3rd | Q4th |  | Q1st | Q2nd | Q3rd | Q4th |  | Q1st | Q2nd | Q3rd | Q4th |
| 1 | Sildenafil citrate | 12.67 | 11.99 | 10.27 | 11.64 |  | 10.96 | 9.59 | 10.27 | 13.01 |  | 14.38 | 16.1 | 16.78 | 16.44 |  | 19.52 | 18.84 | 19.52 | 16.78 |  | 17.47 | 19.86 | 18.15 | 17.47 |
| 2 | Tadalafil | 5.14 | 6.51 | 4.79 | 5.14 |  | 6.51 | 11.3 | 13.7 | 9.93 |  | 13.7 | 11.99 | 12.67 | 13.01 |  | 13.01 | 14.04 | 13.36 | 13.36 |  | 17.12 | 16.44 | 16.44 | 19.86 |
| 3 | Vardenafil hydrochloride | 0.00 | 0.00 | 0.00 | 0.00 |  | 0.00 | 0.00 | 0.00 | 0.00 |  | 0.00 | 0.68 | 0.34 | 0.68 |  | 0.68 | 0.68 | 0.34 | 1.37 |  | 1.03 | 1.03 | 1.03 | 0.34 |
| 4 | Riociguat | 0.34 | 0.34 | 0.00 | 0.00 |  | 0.00 | 0.34 | 0.00 | 0.00 |  | 0.68 | 0.34 | 1.03 | 1.37 |  | 2.05 | 2.74 | 1.71 | 2.05 |  | 1.71 | 2.05 | 2.05 | 2.74 |
| 5 | Ambrisentan | 1.03 | 1.03 | 0.68 | 1.03 |  | 0.68 | 5.48 | 3.42 | 3.08 |  | 4.11 | 6.16 | 5.82 | 6.85 |  | 6.85 | 9.25 | 7.53 | 6.51 |  | 8.22 | 8.90 | 7.53 | 9.93 |
| 6 | Bosentan | 1.03 | 1.03 | 2.05 | 1.03 |  | 1.37 | 2.74 | 2.4 | 3.08 |  | 2.40 | 2.74 | 4.45 | 3.08 |  | 2.74 | 3.42 | 4.11 | 3.08 |  | 2.05 | 2.40 | 2.74 | 1.71 |
| 7 | Macitentan | 0.00 | 0.00 | 0.00 | 0.00 |  | 0.00 | 0.34 | 1.71 | 1.37 |  | 2.05 | 3.42 | 2.74 | 3.08 |  | 3.08 | 3.77 | 4.45 | 4.11 |  | 5.14 | 5.48 | 4.45 | 4.79 |
| 8 | Beraprost Sodium | 28.77 | 31.16 | 30.48 | 32.88 |  | 30.82 | 33.22 | 33.22 | 35.27 |  | 35.62 | 34.93 | 36.3 | 37.33 |  | 38.36 | 39.38 | 38.01 | 36.64 |  | 36.3 | 37.33 | 36.99 | 38.01 |
| 9 | Treprostinil | 0.34 | 0.34 | 0.34 | 0.34 |  | 0.00 | 0.68 | 0.00 | 0.34 |  | 0.68 | 0.00 | 0.00 | 0.34 |  | 0.34 | 0.00 | 0.00 | 0.00 |  | 0.68 | 0.68 | 0.34 | 1.71 |
| 10 | Iloprost | 0.00 | 0.00 | 0.00 | 0.00 |  | 0.00 | 0.00 | 0.00 | 0.00 |  | 0.00 | 0.00 | 0.00 | 0.00 |  | 0.34 | 0.34 | 0.00 | 0.00 |  | 0.34 | 0.00 | 0.34 | 0.00 |
| 11 | Selexipag | 0.00 | 0.00 | 0.00 | 0.00 |  | 0.00 | 0.00 | 0.34 | 0.34 |  | 0.68 | 1.37 | 2.74 | 2.05 |  | 2.40 | 2.74 | 2.40 | 2.40 |  | 2.40 | 1.37 | 2.74 | 1.71 |

Supplementary Table 4A.Monthly Cost of PAH-Targeted Medications as a Percentage of Household Capacity-to-Pay Among the Highest 20% Income Group (Urban vs. Rural, 2019–2023)

| **Name** | **Urban (%)** | | | | | **Rural (%)** | | | | |
| --- | --- | --- | --- | --- | --- | --- | --- | --- | --- | --- |
|  | **2019** | **2020** | **2021** | **2022** | **2023** | **2019** | **2020** | **2021** | **2022** | **2023** |
| Sildenafil citrate | 6.71 | 2.40 | 0.83 | 0.61 | 0.50 | 17.06 | 5.99 | 1.97 | 1.41 | 1.11 |
| Tadalafil | **21.03** | 11.92 | 8.68 | 8.44 | 5.26 | **53.48** | **29.74** | **20.68** | 19.64 | 11.60 |
| Vardenafil hydrochloride | 0.00 | 0.00 | 4.05 | 3.90 | 3.81 | 0.00 | 0.00 | 9.64 | 9.07 | 8.41 |
| Riociguat | **36.77** | 4.25 | 4.02 | 3.84 | 3.79 | **93.51** | 10.61 | 9.57 | 8.93 | 8.36 |
| Ambrisentan | 17.05 | 4.96 | 2.04 | 1.32 | 1.19 | **43.35** | 12.37 | 4.86 | 3.07 | 2.63 |
| Bosentan | 18.23 | 4.73 | 4.48 | 4.30 | 4.16 | **46.37** | 11.79 | 10.67 | 10.01 | 9.18 |
| Macitentan | **130.80** | 6.03 | 5.65 | 5.41 | 5.24 | **332.65** | 15.05 | 13.45 | 12.58 | 11.56 |
| Beraprost Sodium | 4.72 | 4.46 | 4.12 | 3.89 | 3.58 | 12.00 | 11.13 | 9.81 | 9.05 | 7.90 |
| Treprostinil | **278.15** | **267.55** | **250.20** | **236.81** | **40.20** | **707.42** | **667.21** | **595.84** | **551.10** | **88.72** |
| Iloprost | **476.63** | **458.77** | **433.50** | **428.06** | **402.26** | **1212.19** | **1144.07** | **1032.35** | **996.16** | **887.71** |
| Selexipag | 0.00 | 19.22 | 17.79 | 14.89 | 14.55 | 0.00 | **47.94** | **42.38** | **34.66** | **32.11** |

Bold values indicate that the monthly drug cost exceeds 20% of household capacity-to-pay, representing catastrophic health expenditure.

**Supplementary Table 4B.Monthly Cost of PAH-Targeted Medications as a Percentage of Household Capacity-to-Pay Among the Upper-middle 20% Income Group (Urban vs. Rural, 2019–2023**

| **Name** | **Urban (%)** | | | | | **Rural (%)** | | | | |
| --- | --- | --- | --- | --- | --- | --- | --- | --- | --- | --- |
|  | **2019** | **2020** | **2021** | **2022** | **2023** | **2019** | **2020** | **2021** | **2022** | **2023** |
| Sildenafil citrate | 11.62 | 4.20 | 1.43 | 1.05 | 0.85 | **31.16** | 11.04 | 3.65 | 2.64 | 2.14 |
| Tadalafil | **36.44** | **20.86** | 15.10 | 14.66 | 8.89 | **97.71** | **54.85** | **38.45** | **36.72** | **22.39** |
| Vardenafil hydrochloride | 0.00 | 0.00 | 7.04 | 6.77 | 6.44 | 0.00 | 0.00 | 17.94 | 16.96 | 16.23 |
| Riociguat | **63.71** | 7.44 | 6.99 | 6.67 | 6.41 | **170.83** | 19.56 | 17.80 | 16.70 | 16.13 |
| Ambrisentan | **29.54** | 8.68 | 3.55 | 2.29 | 2.01 | **79.20** | **22.82** | 9.04 | 5.74 | 5.07 |
| Bosentan | **31.59** | 8.27 | 7.79 | 7.47 | 7.03 | **84.71** | **21.75** | 19.85 | 18.72 | 17.72 |
| Macitentan | **226.66** | 10.56 | 9.82 | 9.39 | 8.86 | **607.73** | **27.75** | **25.02** | **23.52** | **22.30** |
| Beraprost Sodium | 8.18 | 7.81 | 7.16 | 6.76 | 6.06 | **21.93** | **20.54** | 18.23 | 16.92 | 15.25 |
| Treprostinil | **482.01** | **468.06** | **435.04** | **411.38** | **67.98** | **1292.39** | **1230.63** | **1108.02** | **1030.27** | **171.20** |
| Iloprost | **825.95** | **802.58** | **753.75** | **743.61** | **680.21** | **2214.56** | **2110.17** | **1919.74** | **1862.29** | **1713.02** |
| Selexipag | 0.00 | **33.63** | **30.94** | **25.87** | **24.60** | 0.00 | **88.42** | **78.80** | **64.80** | **61.96** |

Bold values indicate that the monthly drug cost exceeds 20% of household capacity-to-pay, representing catastrophic health expenditure.

**Supplementary Table 4C.Monthly Cost of PAH-Targeted Medications as a Percentage of Household Capacity-to-Pay Among the Middle 20% Income Group (Urban vs. Rural, 2019–2023)**

| **Name** | **Urban (%)** | | | | | **Rural (%)** | | | | |
| --- | --- | --- | --- | --- | --- | --- | --- | --- | --- | --- |
|  | **2019** | **2020** | **2021** | **2022** | **2023** | **2019** | **2020** | **2021** | **2022** | **2023** |
| Sildenafil citrate | 16.23 | 5.87 | 1.99 | 1.47 | 1.20 | **43.97** | 15.67 | 5.12 | 3.73 | 3.00 |
| Tadalafil | **50.90** | **29.16** | **20.96** | **20.44** | 12.57 | **137.87** | **77.86** | **53.84** | **51.86** | **31.48** |
| Vardenafil hydrochloride | 0.00 | 0.00 | 9.78 | 9.44 | 9.11 | 0.00 | 0.00 | **25.11** | **23.95** | **22.81** |
| Riociguat | **89.00** | 10.40 | 9.70 | 9.29 | 9.06 | **241.05** | **27.77** | **24.92** | **23.58** | **22.68** |
| Ambrisentan | **41.26** | 12.13 | 4.93 | 3.19 | 2.85 | **111.76** | **32.39** | 12.65 | 8.10 | 7.13 |
| Bosentan | **44.13** | 11.57 | 10.82 | 10.42 | 9.95 | **119.53** | **30.88** | **27.79** | **26.43** | **24.91** |
| Macitentan | **316.61** | 14.76 | 13.64 | 13.09 | 12.52 | **857.54** | **39.40** | **35.03** | **33.21** | **31.36** |
| Beraprost Sodium | 11.42 | 10.92 | 9.94 | 9.42 | 8.56 | **30.94** | **29.15** | **25.53** | **23.90** | **21.45** |
| Treprostinil | **673.30** | **654.34** | **604.03** | **573.41** | **96.12** | **1823.62** | **1746.99** | **1551.39** | **1455.08** | **240.70** |
| Iloprost | **1153.73** | **1121.99** | **1046.52** | **1036.48** | **961.75** | **3124.85** | **2995.58** | **2687.91** | **2630.19** | **2408.47** |
| Selexipag | 0.00 | **47.01** | **42.96** | **36.07** | **34.79** | 0.00 | **125.52** | **110.34** | **91.52** | **87.12** |

Bold values indicate that the monthly drug cost exceeds 20% of household capacity-to-pay, representing catastrophic health expenditure.

**Supplementary Table 4D.Monthly Cost of PAH-Targeted Medications as a Percentage of Household Capacity-to-Pay Among the Lower-middle 20% Income Group (Urban vs. Rural, 2019–2023)**

| **Name** | **Urban (%)** | | | | | **Rural (%)** | | | | |
| --- | --- | --- | --- | --- | --- | --- | --- | --- | --- | --- |
|  | **2019** | **2020** | **2021** | **2022** | **2023** | **2019** | **2020** | **2021** | **2022** | **2023** |
| Sildenafil citrate | **22.96** | 8.38 | 2.81 | 2.09 | 1.72 | **63.04** | **22.19** | 7.31 | 5.43 | 4.31 |
| Tadalafil | **71.99** | **41.65** | **29.56** | **29.02** | **18.07** | **197.67** | **110.23** | **76.89** | **75.63** | **45.23** |
| Vardenafil hydrochloride | 0.00 | 0.00 | 13.79 | 13.40 | 13.09 | 0.00 | 0.00 | **35.86** | **34.93** | **32.77** |
| Riociguat | **125.86** | 14.85 | 13.68 | 13.20 | 13.01 | **345.59** | **39.31** | **35.59** | **34.40** | **32.58** |
| Ambrisentan | **58.35** | 17.33 | 6.95 | 4.54 | 4.09 | **160.23** | **45.86** | 18.07 | 11.82 | 10.24 |
| Bosentan | **62.41** | 16.52 | 15.26 | 14.79 | 14.29 | **171.36** | **43.72** | **39.69** | **38.55** | **35.78** |
| Macitentan | **447.74** | **21.08** | 19.23 | 18.59 | 17.99 | **1229.44** | **55.78** | **50.03** | **48.44** | **45.04** |
| Beraprost Sodium | 16.15 | 15.59 | 14.02 | 13.38 | 12.31 | **44.36** | **41.27** | **36.46** | **34.85** | **30.81** |
| Treprostinil | **952.15** | **934.55** | **851.90** | **814.38** | **138.13** | **2614.49** | **2473.26** | **2215.63** | **2122.15** | **345.77** |
| Iloprost | **1631.54** | **1602.48** | **1475.98** | **1472.07** | **1382.09** | **4480.03** | **4240.92** | **3838.77** | **3835.97** | **3459.74** |
| Selexipag | 0.00 | **67.15** | **60.59** | **51.22** | **49.99** | 0.00 | **177.70** | **157.58** | **133.48** | **125.15** |

Bold values indicate that the monthly drug cost exceeds 20% of household capacity-to-pay, representing catastrophic health expenditure.

Supplementary Table 4E.Monthly Cost of PAH-Targeted Medications as a Percentage of Household Capacity-to-Pay Among the Lowest 20% Income Group (Urban vs. Rural, 2019–2023)

| **Name** | **Urban (%)** | | | | | **Rural (%)** | | | | |
| --- | --- | --- | --- | --- | --- | --- | --- | --- | --- | --- |
|  | **2019** | **2020** | **2021** | **2022** | **2023** | **2019** | **2020** | **2021** | **2022** | **2023** |
| Sildenafil citrate | **39.55** | 14.78 | 5.06 | 3.83 | 3.18 | **144.26** | **49.25** | 17.44 | 12.94 | 10.54 |
| Tadalafil | **123.99** | **73.44** | **53.20** | **53.32** | **33.29** | **452.32** | **244.69** | **183.45** | **180.10** | **110.52** |
| Vardenafil hydrochloride | 0.00 | 0.00 | **24.81** | **24.63** | **24.12** | 0.00 | 0.00 | **85.57** | **83.18** | **80.09** |
| Riociguat | **216.79** | **26.19** | **24.62** | **24.25** | **23.98** | **790.82** | **87.26** | **84.91** | **81.91** | **79.61** |
| Ambrisentan | **100.51** | **30.55** | 12.50 | 8.33 | 7.54 | **366.65** | **101.80** | **43.12** | **28.14** | **25.02** |
| Bosentan | **107.50** | **29.13** | **27.46** | **27.18** | **26.33** | **392.13** | **97.05** | **94.69** | **91.80** | **87.43** |
| Macitentan | **771.22** | **37.16** | **34.61** | **34.15** | **33.15** | **2813.33** | **123.81** | **119.36** | **115.35** | **110.08** |
| Beraprost Sodium | **27.83** | **27.50** | **25.23** | **24.57** | **22.67** | **101.51** | **91.61** | **86.99** | **83.00** | **75.28** |
| Treprostinil | **1640.06** | **1647.75** | **1532.94** | **1496.23** | **254.49** | **5982.76** | **5489.99** | **5286.33** | **5053.53** | **844.98** |
| Iloprost | **2810.31** | **2825.41** | **2655.95** | **2704.57** | **2546.41** | **10251.69** | **9413.73** | **9159.00** | **9134.69** | **8454.81** |
| Selexipag | 0.00 | **118.39** | **109.02** | **94.11** | **92.11** | 0.00 | **394.44** | **375.97** | **317.85** | **305.83** |

Bold values indicate that the monthly drug cost exceeds 20% of household capacity-to-pay, representing catastrophic health expenditure.

Supplementary Table 5A. Monthly drug cost as percentage of household capacity-to-pay in China.（under 40% reimbursement scenario）

| **Name** | **Total (%)** | | | | | **Urban (%)** | | | | | **Rural (%)** | | | | |
| --- | --- | --- | --- | --- | --- | --- | --- | --- | --- | --- | --- | --- | --- | --- | --- |
|  | **2019** | **2020** | **2021** | **2022** | **2023** | **2019** | **2020** | **2021** | **2022** | **2023** | **2019** | **2020** | **2021** | **2022** | **2023** |
| Sildenafil citrate | 1.67 | 0.6 | 0.2 | 0.15 | 0.12 | 1.21 | 0.44 | 0.15 | 0.11 | 0.09 | 3.2 | 1.12 | 0.37 | 0.27 | 0.21 |
| Tadalafil | 5.23 | 2.97 | 2.11 | 2.04 | 1.24 | 3.79 | 2.18 | 1.57 | 1.53 | 0.94 | 10.03 | 5.57 | 3.92 | 3.75 | 2.24 |
| Vardenafil hydrochloride | 0 | 0 | 0.99 | 0.94 | 0.9 | 0 | 0 | 0.73 | 0.71 | 0.68 | 0 | 0 | 1.83 | 1.73 | 1.62 |
| Riociguat | 9.14 | 1.21 | 1.12 | 1.06 | 1.02 | 6.63 | 0.89 | 0.83 | 0.8 | 0.77 | 17.53 | 2.27 | 2.07 | 1.95 | 1.84 |
| Ambrisentan | 4.24 | 1.23 | 0.41 | 0.37 | 0.32 | 3.07 | 0.91 | 0.3 | 0.27 | 0.24 | 8.13 | 2.32 | 0.76 | 0.67 | 0.58 |
| Bosentan | 4.53 | 1.34 | 1.25 | 1.19 | 1.12 | 3.29 | 0.99 | 0.92 | 0.89 | 0.85 | 8.69 | 2.53 | 2.31 | 2.18 | 2.02 |
| Macitentan | **32.52** | 1.71 | 1.57 | 1.5 | 1.41 | **23.59** | 1.26 | 1.16 | 1.12 | 1.06 | **62.38** | 3.22 | 2.92 | 2.74 | 2.54 |
| Beraprost Sodium | 1.17 | 1.11 | 1 | 0.94 | 0.84 | 0.85 | 0.82 | 0.74 | 0.71 | 0.64 | 2.25 | 2.09 | 1.86 | 1.73 | 1.52 |
| Treprostinil | **69.15** | **66.54** | **60.9** | **57.37** | 9.01 | **50.17** | **48.86** | **45.12** | **42.94** | 6.82 | **132.65** | **125.02** | **113** | **105.1** | 16.29 |
| Iloprost | **118.49** | **114.09** | **105.51** | **103.7** | **94.57** | **85.97** | **83.78** | **78.17** | **77.61** | **71.57** | **227.3** | **214.38** | **195.78** | **189.98** | **170.99** |
| Selexipag | 0 | 5.46 | 4.95 | 4.12 | 3.91 | 0 | 4.01 | 3.67 | 3.09 | 2.96 | 0 | 10.27 | 9.18 | 7.55 | 7.07 |

Bold values indicate that the monthly drug cost exceeds 20% of household capacity-to-pay, representing catastrophic health expenditure.

Supplementary Table 5B. Monthly drug cost as percentage of household capacity-to-pay in China.（under 90% reimbursement scenario）

| **Name** | **Total (%)** | | | | | **Urban (%)** | | | | | **Rural (%)** | | | | |
| --- | --- | --- | --- | --- | --- | --- | --- | --- | --- | --- | --- | --- | --- | --- | --- |
|  | **2019** | **2020** | **2021** | **2022** | **2023** | **2019** | **2020** | **2021** | **2022** | **2023** | **2019** | **2020** | **2021** | **2022** | **2023** |
| Sildenafil citrate | 1.67 | 0.60 | 0.20 | 0.15 | 0.12 | 1.21 | 0.44 | 0.15 | 0.11 | 0.09 | 3.20 | 1.12 | 0.37 | 0.27 | 0.21 |
| Tadalafil | 5.23 | 2.97 | 2.11 | 2.04 | 1.24 | 3.79 | 2.18 | 1.57 | 1.53 | 0.94 | 10.03 | 5.57 | 3.92 | 3.75 | 2.24 |
| Vardenafil hydrochloride | 0.00 | 0.00 | 0.99 | 0.94 | 0.90 | 0.00 | 0.00 | 0.73 | 0.71 | 0.68 | 0.00 | 0.00 | 1.83 | 1.73 | 1.62 |
| Riociguat | 9.14 | 0.30 | 0.28 | 0.27 | 0.25 | 6.63 | 0.22 | 0.21 | 0.20 | 0.19 | 17.53 | 0.57 | 0.52 | 0.49 | 0.46 |
| Ambrisentan | 4.24 | 1.23 | 0.10 | 0.09 | 0.08 | 3.07 | 0.91 | 0.08 | 0.07 | 0.06 | 8.13 | 2.32 | 0.19 | 0.17 | 0.14 |
| Bosentan | 4.53 | 0.34 | 0.31 | 0.30 | 0.28 | 3.29 | 0.25 | 0.23 | 0.22 | 0.21 | 8.69 | 0.63 | 0.58 | 0.55 | 0.51 |
| Macitentan | **32.52** | 0.43 | 0.39 | 0.37 | 0.35 | 23.59 | 0.31 | 0.29 | 0.28 | 0.27 | **62.38** | 0.81 | 0.73 | 0.69 | 0.64 |
| Beraprost Sodium | 1.17 | 1.11 | 1.00 | 0.94 | 0.84 | 0.85 | 0.82 | 0.74 | 0.71 | 0.64 | 2.25 | 2.09 | 1.86 | 1.73 | 1.52 |
| Treprostinil | **69.15** | **66.54** | **60.90** | **57.37** | 2.25 | **50.17** | **48.86** | **45.12** | **42.94** | 1.71 | **132.65** | **125.02** | **113.00** | **105.10** | 4.07 |
| Iloprost | **118.49** | **114.09** | **105.51** | **103.70** | **94.57** | **85.97** | **83.78** | **78.17** | **77.61** | **71.57** | **227.30** | **214.38** | **195.78** | **189.98** | **170.99** |
| Selexipag | 0.00 | 1.37 | 1.24 | 1.03 | 0.98 | 0.00 | 1.00 | 0.92 | 0.77 | 0.74 | 0.00 | 2.57 | 2.30 | 1.89 | 1.77 |

Bold values indicate that the monthly drug cost exceeds 20% of household capacity-to-pay, representing catastrophic health expenditure.
